# Supplementary material for: Chromatin-accessibility estimation from single-cell ATAC-seq data with scOpen
Source: Nat Commun. 2021 Nov 4;12:6386. doi: 10.1038/s41467-021-26530-2 (PMC8568974; doi:10.1038/s41467-021-26530-2)
Supplement: Supplementary file 1 — Supplementary Information [file 41467_2021_26530_MOESM1_ESM.pdf]

# Chromatin-accessibility estimation from single-cell ATAC-seq data with scOpen

Zhijian Li[1, +], Christoph Kuppe[2,3 +], Susanne Ziegler[2], Mingbo Cheng[1], Nazanin Kabgani[2], Sylvia Menzel[2], Martin Zenke[4, 5], Rafael Kramann[2,3,6 *], and Ivan G. Costa[1, *]

[1]Institute for Computational Genomics, Joint Research Center for Computational Biomedicine, RWTH Aachen University Medical School, 52074 Aachen, Germany
[2]Institute of Experimental Medicine and Systems Biology, RWTH Aachen University Medical School, 52074 Aachen, Germany
[3]Division of Nephrology and Clinical Immunology, RWTH Aachen University, 52074 Aachen, Germany
[4]Department of Cell Biology, Institute of Biomedical Engineering, RWTH Aachen University Medical School, Aachen, 52074, Germany
[5]Helmholtz Institute for Biomedical Engineering, RWTH Aachen University, Aachen, Germany
[6]Department of Internal Medicine, Nephrology and Transplantation, Erasmus Medical Center, 3015GD Rotterdam, The Netherlands
[*]corresponding authors: rkramann@ukaachen.de, ivan.costa@rwth-aachen.de
[+]these authors contributed equally to this work

October 25, 2021

# Supplementary Figures

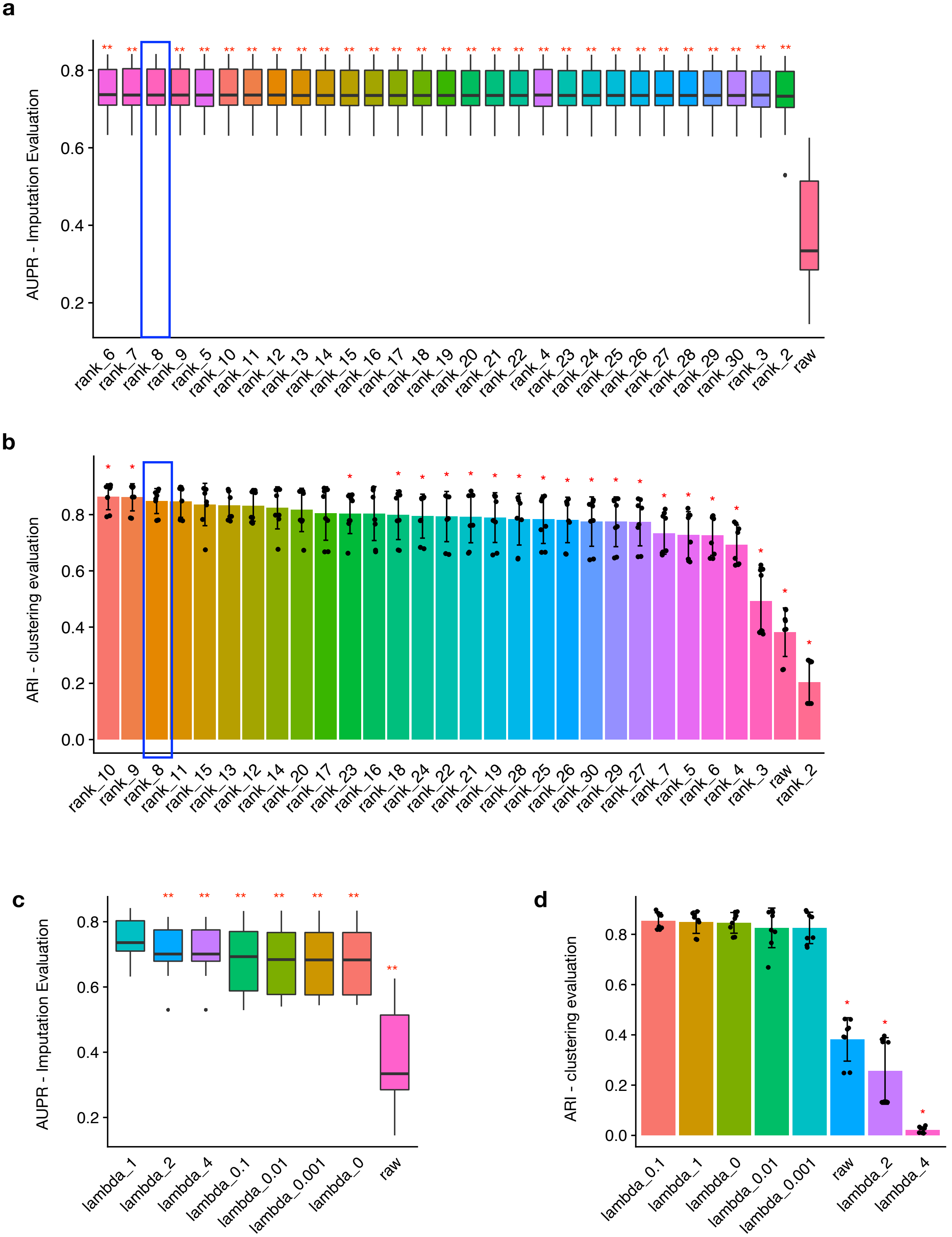

Figure 1: **Evaluation of hyper-parameters in scOpen using simulated dataset.** **a** Boxplot showing the evaluation of imputation/denoising methods for recovering true peaks. The y-axis indicates the area under precision-recall curve (AUPR). Methods are ranked by the mean AUPR. Asterisks denote statistical significance at confident level of 0.95 by comparing rank=8 to other values (** $p < 0.01$, Wilcoxon Rank Sum test, two-tailed) (n = 2,600 cells). **b** Bar plot showing ARI metric for clustering evaluation. Error bars represent the standard deviation of ARI and the center represents the mean value of ARI. Asterisks denote statistical significance at confident level of 0.95 by comparing rank=8 to other values (** $p < 0.01$, Wilcoxon Rank Sum test, two-tailed) (n = 8 independent clustering experiments). **c** Boxplot showing the evaluation of imputation/denoising methods for recovering true peaks. The y-axis indicates the area under precision-recall curve (AUPR). Methods are ranked by the mean AUPR. Asterisks denote statistical significance at confident level of 0.95 by comparing $\lambda = 1$ to other values (** $p < 0.01$ and * $p < 0.05$, Wilcoxon Rank Sum test, two-tailed) (n = 2,600 cells). **d** Same as **c** for ARI metric (clustering evaluation). Error bars represent the standard deviation of AUPR and the center represents the mean value of AUPR. We compared between $\lambda = 1$ and other values (** $p < 0.01$ and * $p < 0.05$ with Wilcoxon Rank Sum test, two-tailed) (n = 8 independent clustering experiments). The box plots in **a** and **c** represent the median (central line), first and third quartiles (box bounds). The whiskers present the 1.5 interquartile range (IQR) and external dots represents outliers (data greater than or smaller than 1.5IQR). Data in **b** and **d** are presented as mean ± SD.

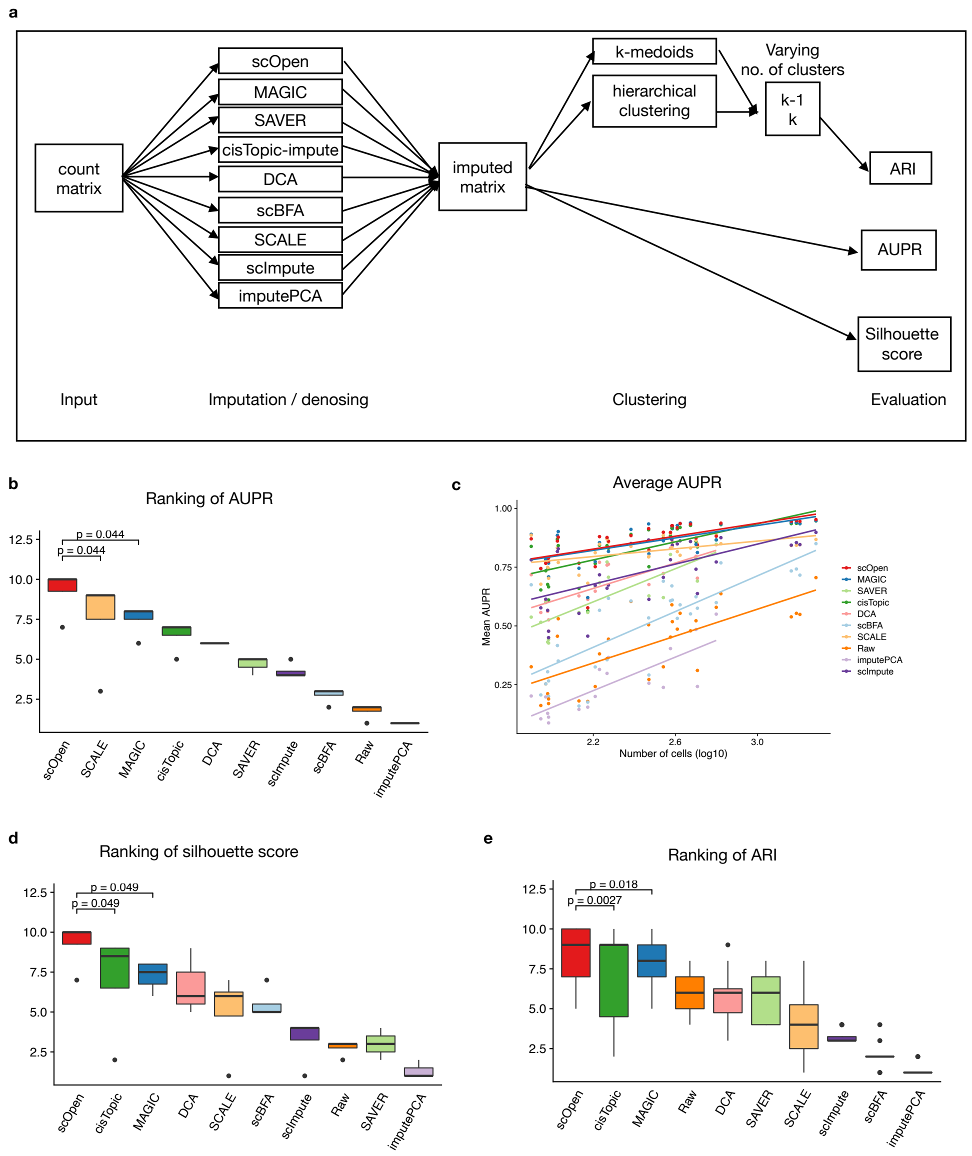


**Supplementary Fig. 2. Evaluation of imputation methods.** **a** Experimental design for benchmarking competing methods. **b** Ranking of imputation methods in terms of average AUPR for each benchmarking dataset. Methods are ordered by median value of ranks. Wilcoxon Rank Sum test (one-sided, paired) was used to compare scOpen with SCALE and MAGIC ($n = 4$ independent datasets). **c** Scatter plot associating the average AUPR and number of cells for each cell type in all benchmarking datasets. Each dot represents a cell type and color refers to method. The x-axis represents the number of cells for each cell type. The trend line was fitted for each method. **d** Ranking of imputation methods using silhouette score as metric for each dataset. Wilcoxon Rank Sum test (one-sided, paired) was used to compare scOpen with cisTopic and MAGIC ($n = 4$ independent datasets). **e** Ranking of methods using ARI as metric for benchmarking datasets. Wilcoxon Rank Sum test (one-sided, paired) was used to compare scOpen with cisTopic and MAGIC ($n = 4$ independent datasets). The box plots in **b**, **d**, and **e** represent the median (central line), first and third quartiles (box bounds). The whiskers present the 1.5IQR and external dots represents outliers (data greater than or smaller than 1.5IQR).

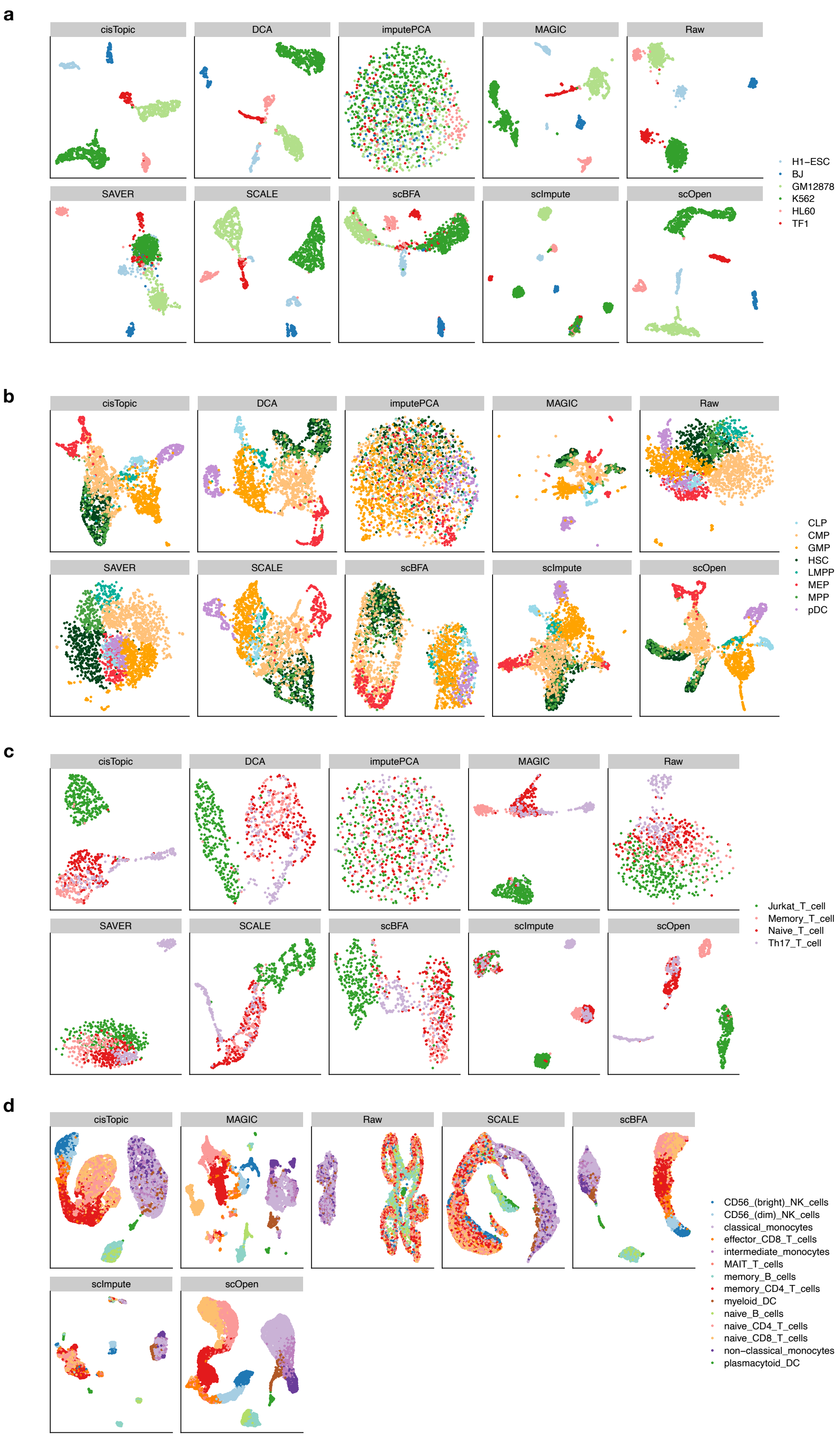


**Supplementary Fig. 3. Visualization of imputation methods on benchmarking datasets.** **a** UMAP embedding of scOpen, cisTopic, DCA, MAGIC, SAVER, scImpute, imputePCA and the raw data for cell line dataset. **b** Same as **a** for Hematopoiesis. **c** Same as **a** for T-cells. **d** Same as **a** for multi-omics PBMC.

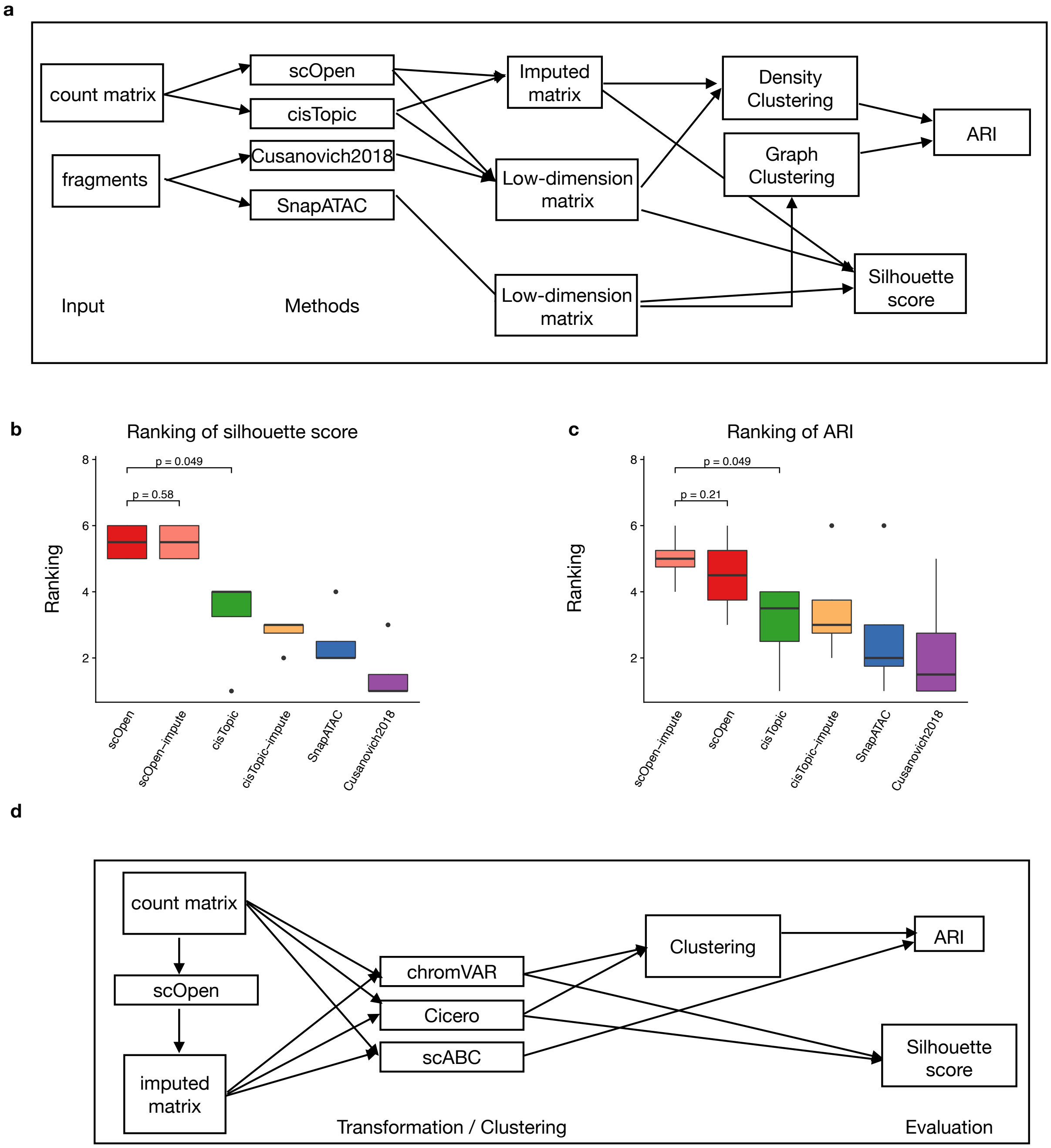


**Supplementary Fig. 4. Experimental design for benchmarking scOpen.** **a** Experimental design for benchmarking of clustering pipelines for scATAC-seq. **b** Boxplot showing the rank of silhouette score for clustering pipelines. We compared the top ranked method (scOpen) with the top-2 runner-up methods using Wilcoxon Rank Sum test (one-sided, paired) ($n = 4$ independent datasets) **c** Same as **b** for ARI. We compared the top ranked method (scOpen) with the top-2 runner-up methods using Wilcoxon Rank Sum test (one-sided, paired) ($n = 4$ independent datasets). **d** Experimental design for benchmarking downstream analysis methods between using raw or scOpen estimated matrix as input. The results were evaluated in terms of clustering with ARI as metric and silhouette score. Clustering was performed using the built-in method in each method. The box plots in **b** and **c** represent the median (central line), first and third quartiles (box bounds). The whiskers present the 1.5IQR and external dots represents outliers (data greater than or smaller than 1.5IQR)

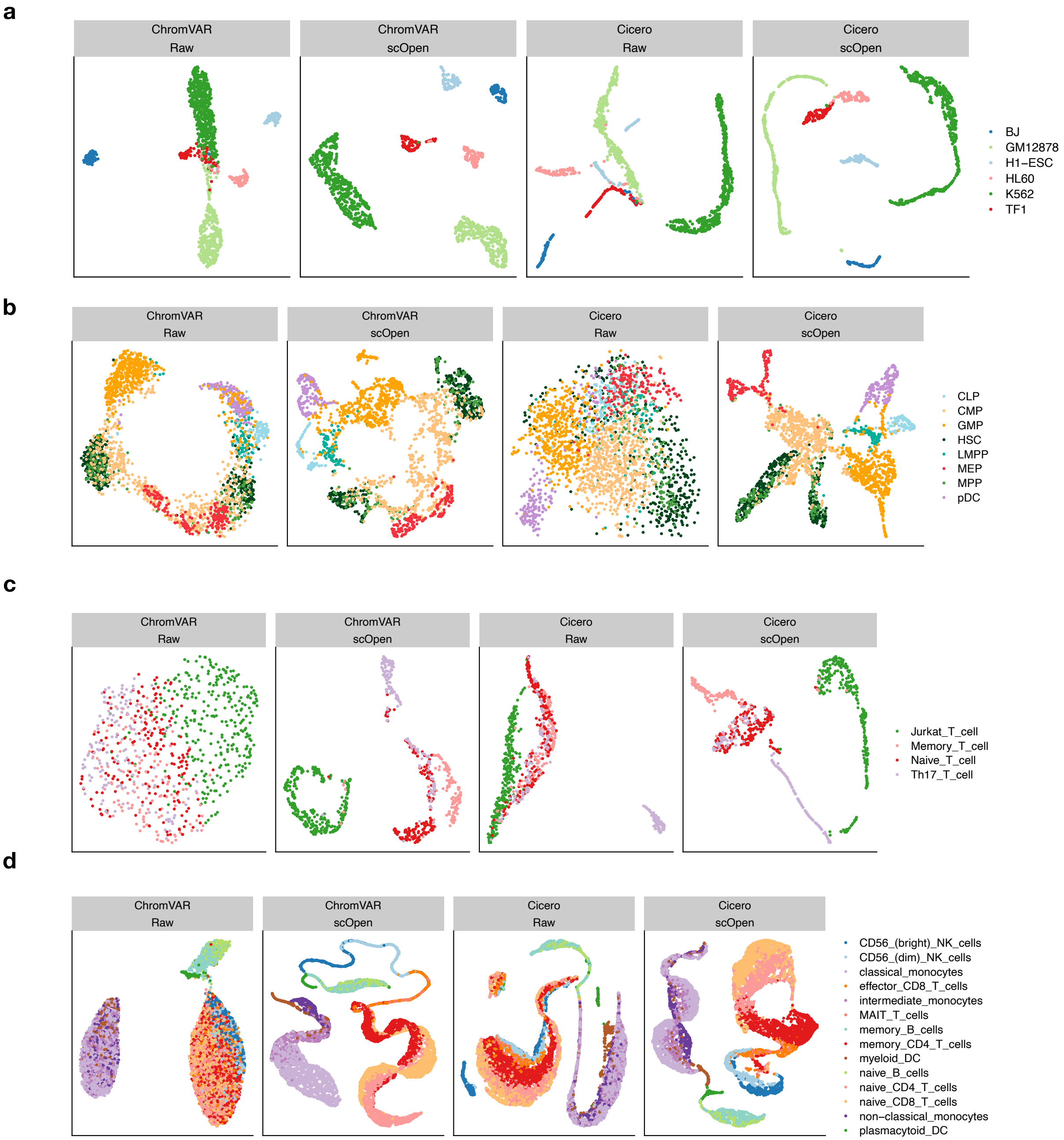


**Supplementary Fig. 5. Benchmarking and visualization for downstream analysis of scATAC-seq. a** UMAP embedding of chromVAR and Cicero transformed data using either raw or scOpen estimated matrix as input for cell line dataset. **b** Same as **a** for Hematopoiesis. **c** Same as **a** for T-cells. **d** Same as **a** for multi-omics PBMC.

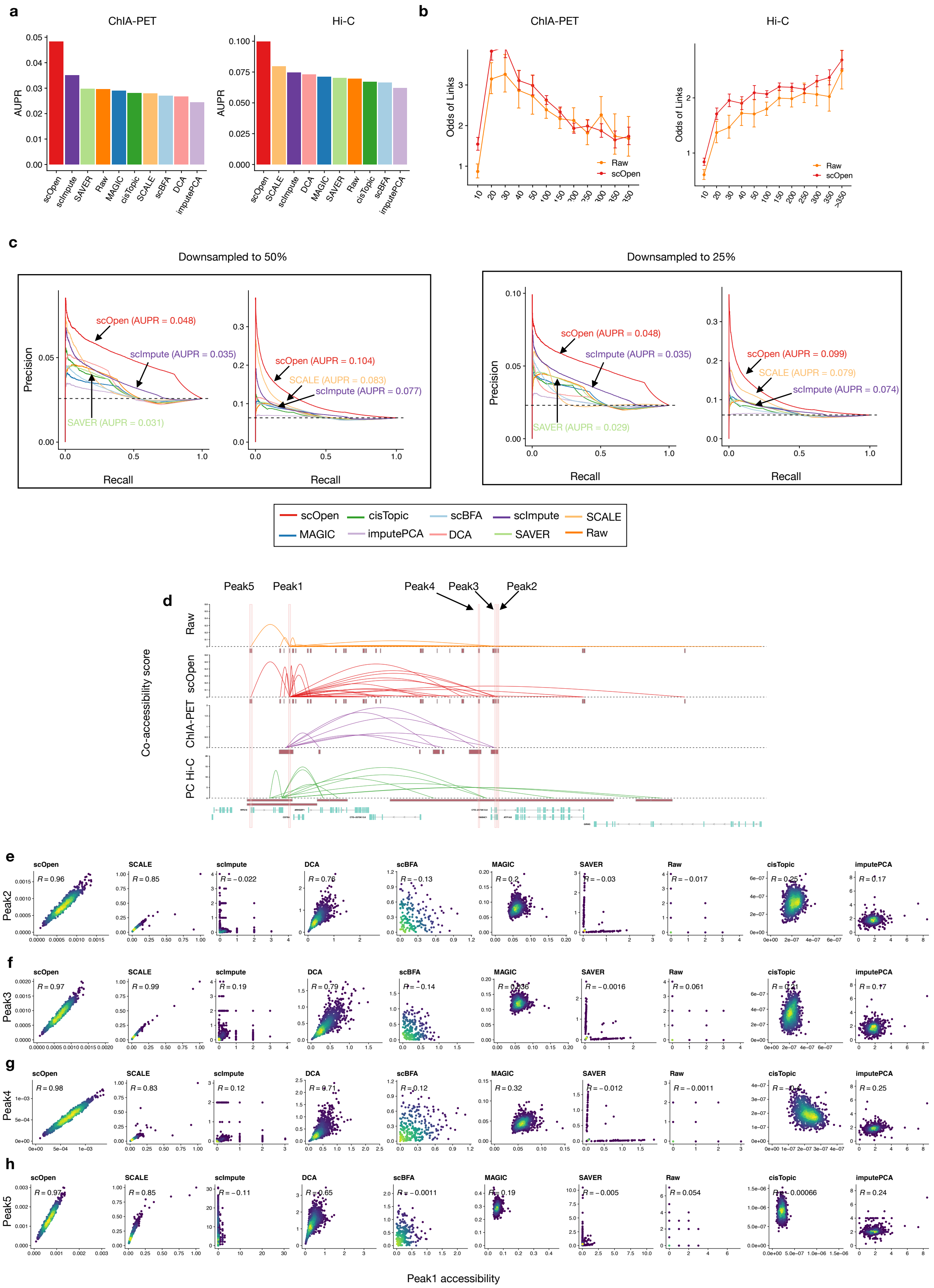
a
ChIA-PET
Hi-C
AUPR
scOpen
scImpute
SAVER
Raw
MAGIC
cisTopic
SCALE
scBFA
DCA
imputePCA
b
ChIA-PET
Hi-C
Odds of Links
Raw
scOpen
c
Downsampled to 50%
Downsampled to 25%
Precision
Recall
scOpen (AUPR = 0.048)
scImpute (AUPR = 0.035)
SAVER (AUPR = 0.031)
scOpen (AUPR = 0.104)
SCALE (AUPR = 0.083)
scImpute (AUPR = 0.077)
scOpen (AUPR = 0.048)
scImpute (AUPR = 0.035)
SAVER (AUPR = 0.029)
scOpen (AUPR = 0.099)
SCALE (AUPR = 0.079)
scImpute (AUPR = 0.074)
scOpen
cisTopic
scBFA
scImpute
SCALE
MAGIC
imputePCA
DCA
SAVER
Raw
d
Peak5
Peak1
Peak4
Peak3
Peak2
Co-accessibility score
Raw
scOpen
ChIA-PET
PC Hi-C
e
f
g
h
Peak2
Peak3
Peak4
Peak5
Peak1 accessibility

Figure 6: **Benchmarking of Cicero predicted links.** **a** Barplots showing AUPR of the predicted peak-peak co-accessibility links using either raw or imputed matrix with H1-ESC single cell ATAC-seq data. Analysis was executed with Cicero. Left, links are evaluated using ChIA-PET data as true labels. Right, links are evaluated using Hi-C data as true labels. **b** Odds ratio ($y$-axis) of Cicero predicted co-accessible sites (n = 3853260) also supported by pol-II ChIA-PET (left) and PC Hi-C (right) vs. distance between sites ($x$-axis). Error bars indicate 95% confidence intervals calculated using Fisher's exact test and the center of error bars represent the mean odds ratio. Odds ratio superior than 1 indicates a positive relationship. **c** Precision-recall curves showing the evaluation of the predicted links on GM12878 cells using the raw and imputed matrix as input after down-sampling to 50% (left) or 25% (right). Related to **Fig. 2e-f**. **d** Visualization of co-accessibility scores (y-axis) of Cicero predicted with raw and scOpen estimated matrices contrasted with scores based on RNA pol-II ChIA-PET (purple) and promoter capture Hi-C (green) around the *CD79A* locus (x-axis). We highlighted more links. Related to **Fig. 2g**. **e** Scatter plots showing single cell accessibility scores from raw data or estimated by imputation methods for peak1-to-peak2 link as shown in **c**. **f** Same as **e** for link peak1-to-peak3. **g** Same as **e** for link peak1-to-peak4. **h** Same as **e** for link peak1-to-peak4.

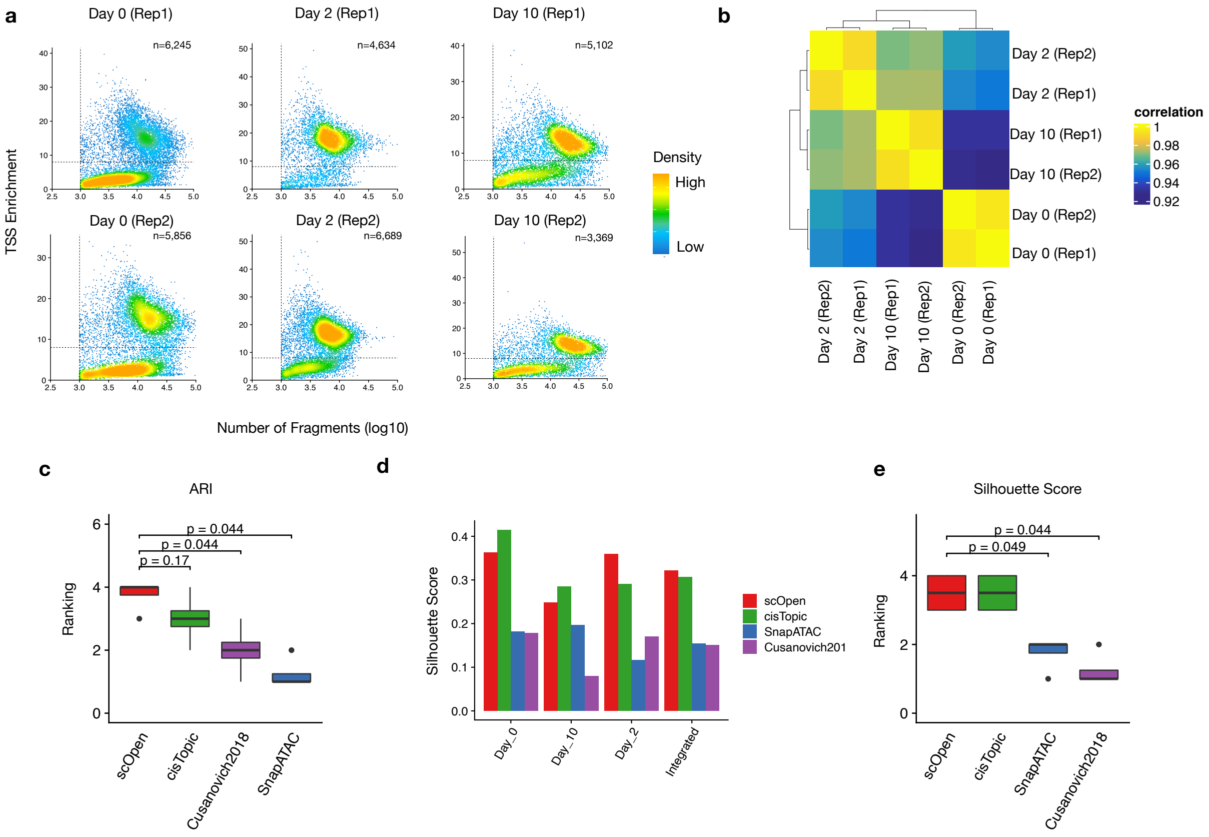


**Supplementary Fig. 7. Quality control and benchmarking of UUO scATAC-seq a** Scatter plot showing number of unique fragments vs. TSS enrichment of UUO scATAC-seq for each sample. Each dot represents a cell and the dash lines represent cut-off used for cell filtering. The number of cells that pass filtering is shown on right-upper corner. **b** Heatmap showing the correlation between samples. **c** Rank of ARI for distinct scATAC-seq dimension reduction/clustering pipelines. Wilcoxon Rank Sum test (one-sided, paired) was used to compare scOpen with SnapATAC and Cusanovich2018 (n = 4 independent experiments). **d** Barplot showing silhouette score of each sample by using distinct dimension reduction/clustering pipelines after data integration and label transferring. **e** Same as **c** for silhouette score. Wilcoxon Rank Sum test (one-sided, paired) was used to compare scOpen with SnapATAC and Cusanovich2018 (n = 4 independent experiments). The box plots in **c** and **e** represent the median (central line), first and third quartiles (box bounds). The whiskers present the 1.5IQR and external dots represents outliers (data greater than or smaller than 1.5IQR)

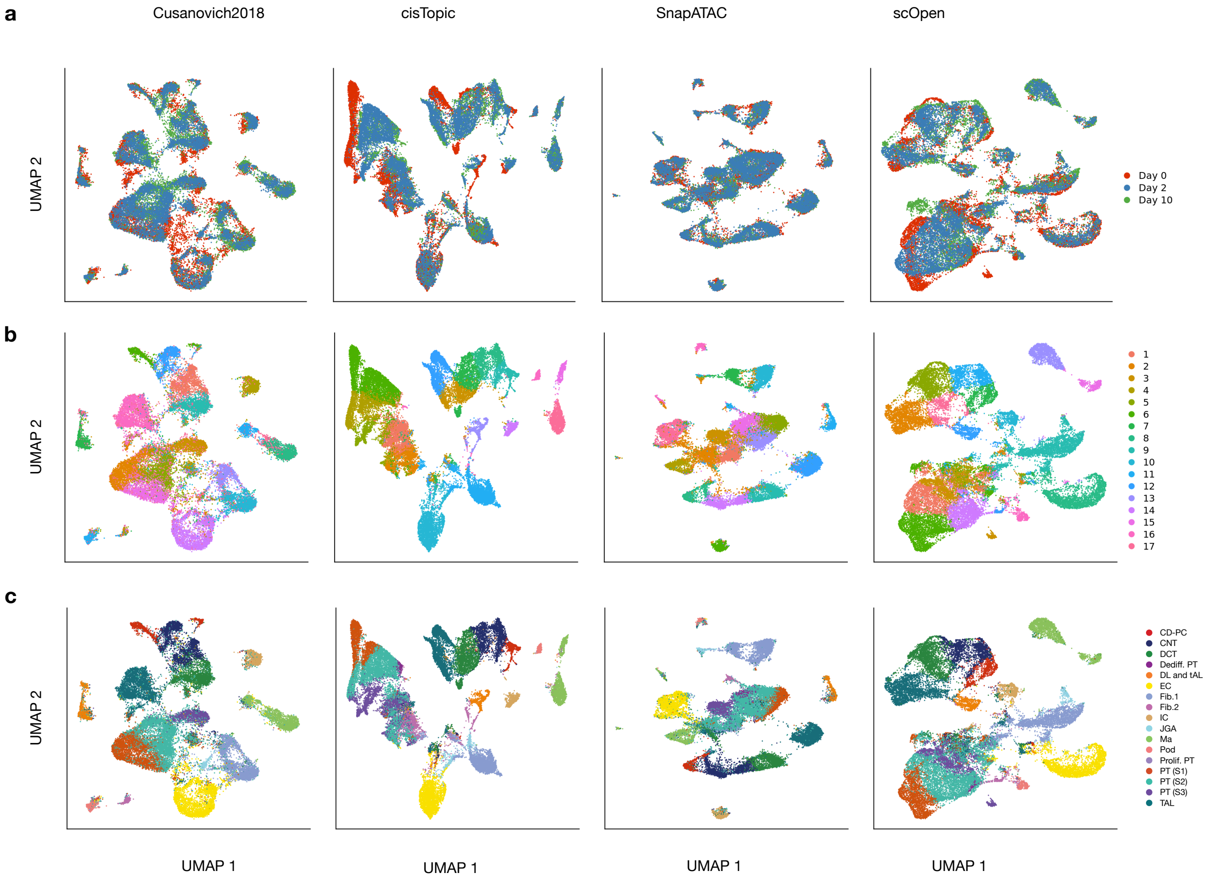


**Supplementary Fig. 8. Visualization of UUO scATAC-seq a** UMAP plots showing data integration results by using different scATAC-seq dimension reduction/clustering pipelines. Each dot represent a cell and cells are colored by time point. **b** UMAP plots showing clustering results for each dimension reduction method. Cells are colored by cluster. **c** UMAP plots showing label transferred results from a public UUO scRNA-seq dataset. Cells are colored by predicted label.

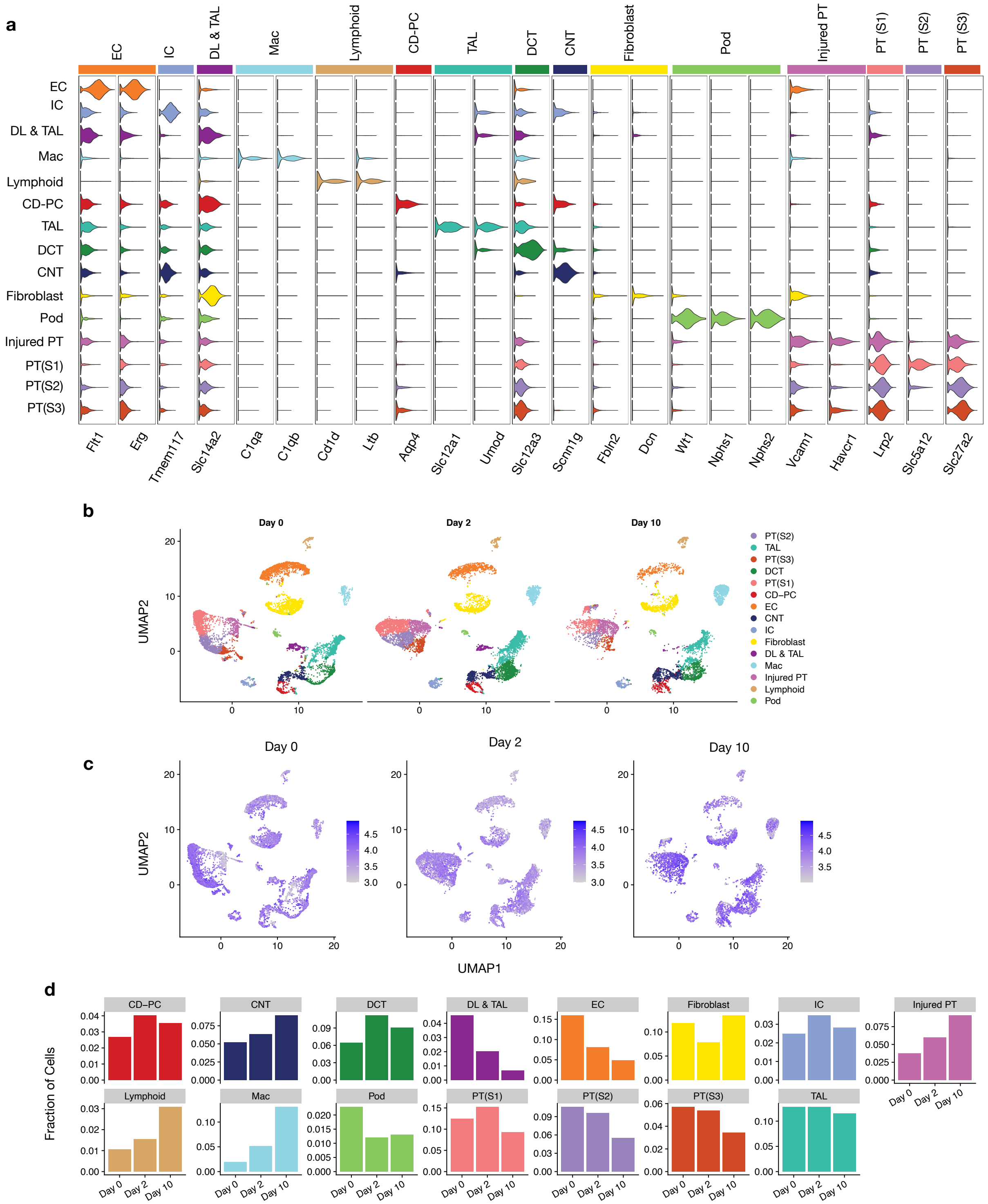


**Supplementary Fig. 9. Visualization of UUO annotation a** Violin plot showing cluster-specific (y-axis) gene accessibility score associated to known marker genes for kidney cells (x-axis). **b** Scatter plots showing condition-specific UMAP visualization of UUO scATAC-seq data for each day. **c** Visualization of data quality for **b**. Colors refer to number of fragments per cell (log10). **d** Bar plots showing proportion of each cell type across different days.

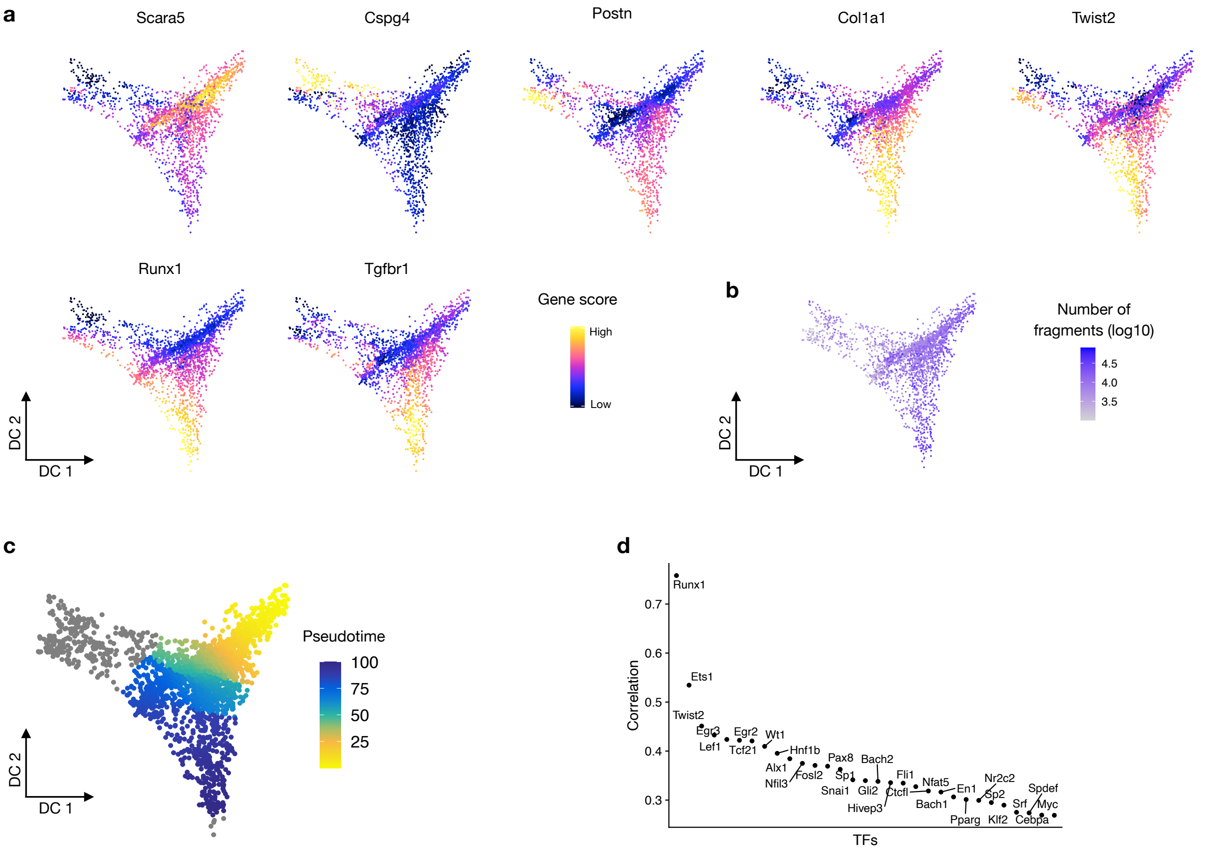


**Supplementary Fig. 10. Identification of TFs driving the differentiation of fibroblasts to myofibroblasts a** Diffusion map embedding of fibroblast showing gene activity score for Scara5 (fibroblast), Cspg4 (pericytes), Postn (myofibroblasts), Col1a1 (myofibroblasts), Twist2, Runx1 and Tgfbr1. **b** Visualization of data quality for **a**. Colors refer to number of fragments (log10) per cell. **c** Visualization of trajectory from fibroblast to myofibroblasts. Colors refer to pseudo-time during of the trajectory. **d** Correlation of gene activity score and motif deviation (associated to **Fig. 4c**) along the trajectory for selected TFs. Each dot represent a TF and TFs are sorted by their correlation. Runx1 has the highest correlation, followed by ETS1 and Twist2. Of these, Runx1 and Twist2 have an increase in gene scores and TF activity .

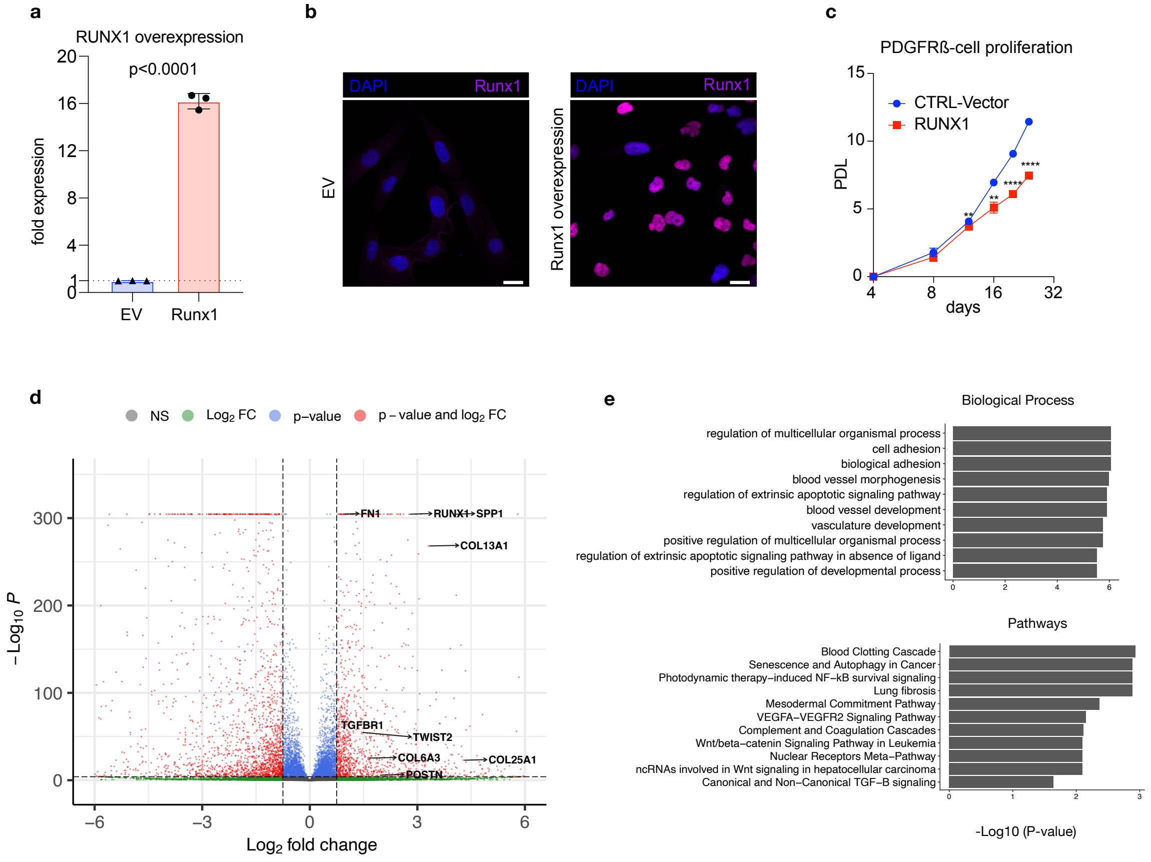


**Supplementary Fig. 11. Validation of Runx1 a** Expression of Runx1 by qPCR after retroviral Runx1 overexpression in a human kidney PDGRFb+ cell line. Error bars represent the SD of the intensity. Data are presented as mean ± SD. Statistical significance was assessed by a two-tailed Student's t-test with $p < 0.05$ being considered statistically significant (n=3). **b** Immuno-fluorescence (IF) staining of Runx1 in human kidney PDGFRb+ cells retrovirally transduced with either empty vector (EV) or an Runx1 overexpression construct (Runx1). Scale bars represent 10 µm. **c** Population doubling of Runx1 overexpressing cells vs. control (EV). Statistical significance was assessed by a two-tailed Student's t-test with $p < 0.05$ being considered statistically significant (n=3). **d** Volcano plot showing differential expression analysis between Runx1 overexpression vs. control. Each dot represents a gene and dashed lines represent the thresholds ($x = 1.5$ and $y = 5$) used for selection of DE genes. Colors refer to significance given different criteria. For details on statistics and reproducibility, see Methods section. **e** Barplot showing GO enrichment using up-regulated genes from Runx1 overexpression. Top 10 terms are shown for Biological Process and WikiPathways.

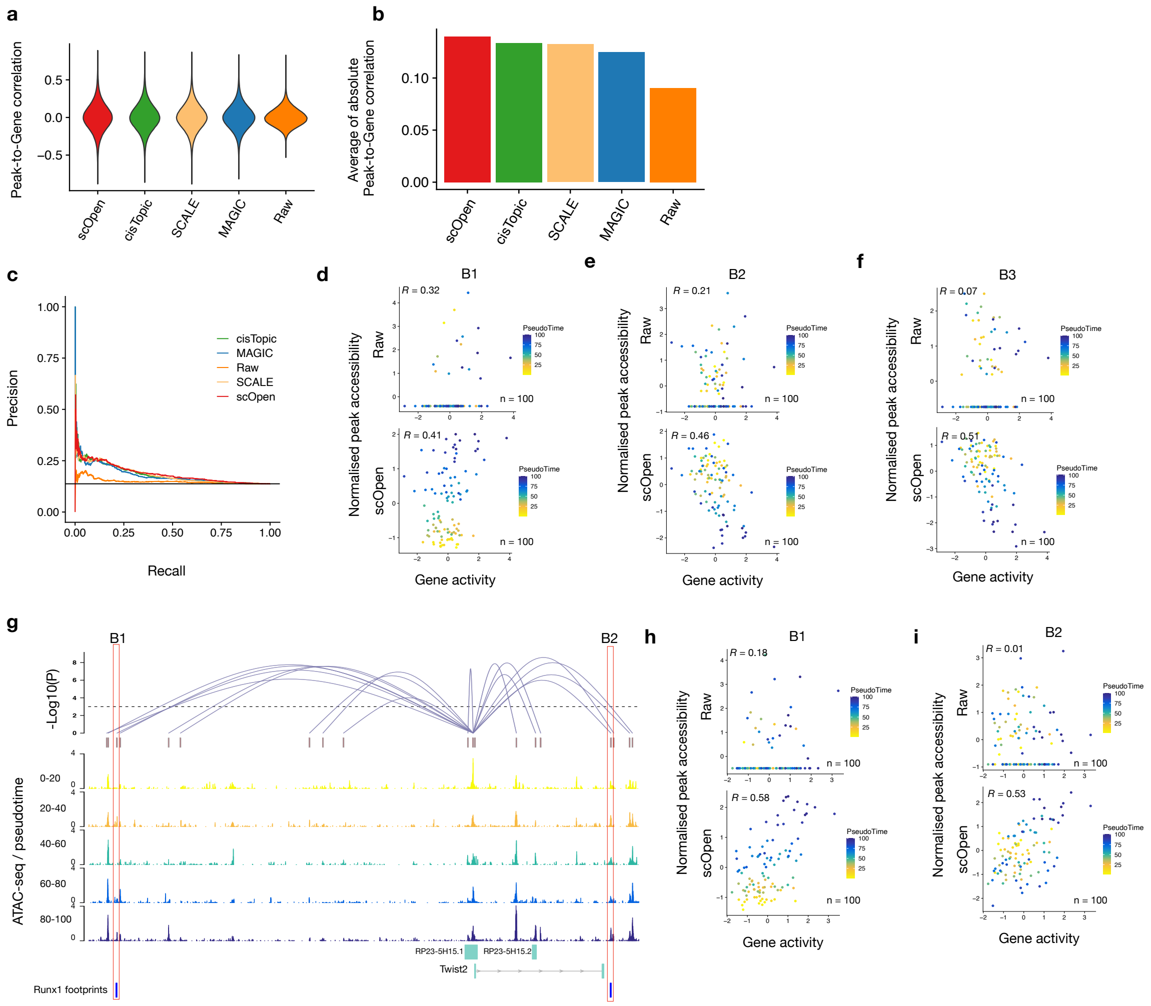


**Supplementary Fig. 12. Visualization and validation of Runx1 target genes a** Violin plot showing predicted Peak-to-Gene (P2G) links using raw or imputed matrix from scOpen, cisTopic, SCALE and MAGIC. Methods are sorted by average absolute value. **b** Average absolute value of correlation of P2G links. **c** AUPR of predicted Runx1 target genes using from raw, scOpen, SCALE or cisTopic matrix. Related to **Fig. 4h**. **d** Scatter plot showing gene activity of Tgfbr1 and linked peak accessibility from raw (upper) or scOpen estimated matrix (lower) for peaks B1 indicated in **Fig. 4i** of the main manuscript. Each dot represents a single cell and the color refers to pseudo-time. The correlation is shown on left-upper corner. Both gene activity score and peak accessibility are z-score normalized. **e** Same as **d** for Peak-to-Gene link B2. **f** Same as **d** for Peak-to-Gene link B3. **g** Peak-to-Gene links of Twist2 in fibroblast cells. Each loop refers to a link and height of loop represent the significance (dash line represents threshold of FDR= 0.001). ATAC-seq tracks were generated from pseudo-bulk profiles of cell along the pseudo-time. Peaks with binding sites of Runx1 supported by ATAC-seq footprints are highlighted (B1-B2). **h** Scatter plot showing gene activity of Twist2 and B1 peak accessibility from raw (upper) or scOpen estimated matrix (lower). **i** Same as **h** for the Peak-to-Gene B2.

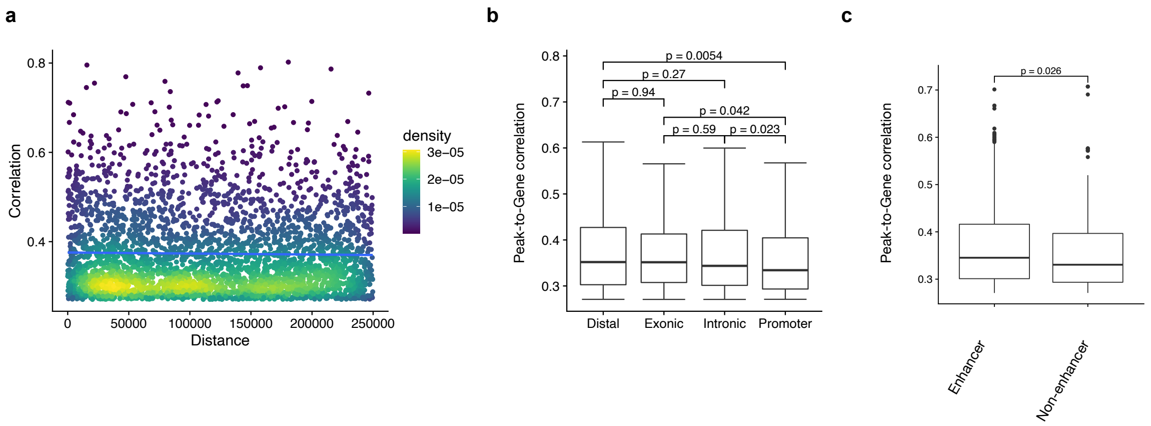


**Supplementary Fig. 13. Statistics of peak-to-gene links a** Scatter plot showing the peak-to-gene correlation and the distance of ATAC-seq peaks to TSS. A line is fitted to show the pattern. **b** Boxplot showing the peak-to-gene correlation distribution. Peaks were annotated as distal, exonic, intronic, and promoter. The Wilcoxon Rank Sum test (two-sided) was used to compare the correlation between different type of peaks (n = 1,196 peaks for Distal, n = 195 peaks for Exonic, n = 1,775 peaks for Intronic, and n = 331 peaks for Promoter). **c** Same as **b**. Peaks were classified as (active) enhancers if overlapping with H3k27Ac and H3K4me1 ChIP-seq peaks from mouse kidneys, otherwise, non-enhancers if only H3K27ac is present. We compared the correlation between enhancers and non-enhancers with Wilcoxon Rank Sum test (two-sided) (n = 728 for Enhancer and n = 299 for Non-enhancer). The box plots in **b** and **c** represent the median (central line), first and third quartiles (box bounds). The whiskers present the 1.5IQR and external dots represents outliers (data greater than or smaller than 1.5IQR)

**Supplementary Tab. 1. Statistics of data sets used in this study**. The number of detected cells, number of regions (peaks), fraction of non-zero entries, average number of reads per cell, fraction of reads in peaks (FRiP) and total number of valid reads are shown below. For comparison purposes, we also included similar statistics for a scRNA-seq data corresponding to the Hematopoiesis scATAC-seq data. We observed a higher sparsity in scATAC-seq (0.039 vs 0.119) despite the fact scATAC-seq data has 7 times more reads per cell than scRNA-seq.

| Dataset | Type | Cells | Features | Non-zeros | Reads per cell | FRIP | Reads | Optimal Rank |
|---|---|---|---|---|---|---|---|---|
| Cell lines | scATAC-seq | 1,224 | 125,647 | 0.036 | 41,467.80 | 0.248 | 50,756,587 | 8 |
| T cells | scATAC-seq | 765 | 49,344 | 0,033 | 14,963.39 | 0.418 | 11,446,993 | 8 |
| Hematopoiesis | scATAC-seq | 2,210 | 109,418 | 0.039 | 34.656.15 | 0.272 | 76,590,091 | 9 |
| Hematopoiesis | scRNA-seq | 14,432 | 12,558 | 0.119 | 5.209,45 | NA | 75,182,840 | NA |
| PBMC | scATAC-seq | 10,032 | 106,935 | 0.067 | 13,486 | 0.714 | 457,001,034 | 7 |
| UUO | scATAC-seq | 31,129 | 150,593 | 0.042 | 13,933 | 0.467 | 419,794,555 | 20 |

**Supplementary Tab. 2. Primers used in this study**

| Genes | Forward prime | Reverse prime |
|---|---|---|
| *RUNX1* | 5‘-CAGTCGACTCTCAACGGCAC | 5‘-TAGGTGAAGGCGCCTGGATA |
| *collagen type I alpha I chain* | 5‘-CCCAGCCACAAAGAGTCTACA | 5‘-ATTGGTGGGATGTCTTCGTCT |
| *fibronectin 1* | 5‘-AACAAACACTAATGTTAATTGCCCA | 5‘-TCGGGAATCTTCTCTGTCAGC |
| *actin alpha 2, smooth muscle* | 5‘-ACTGCCTTGGTGTGTGACAA | 5‘-CACCATCACCCCCTGATGTC |
| *GAPDH* | 5‘-GACAGTCAGCCGCATCTTCT | 5‘-GCGCCCAATACGACCAAATC |
