# Supplementary material for: Chromatin-accessibility estimation from single-cell ATAC-seq data with scOpen
Source: Nat Commun. 2021 Nov 4;12:6386. doi: 10.1038/s41467-021-26530-2 (PMC8568974; doi:10.1038/s41467-021-26530-2)
Supplement: Supplementary file 3 — Description of Additional Supplementary Files [file 41467_2021_26530_MOESM3_ESM.pdf]

## Description of Additional Supplementary Files

**File name: Supplementary Data 1**

Description: The memory and running time requirements of imputation methods on benchmarking datasets.

**File name: Supplementary Data 2**

Description: The complete table for TF activity score across the selected cell types.
