# Supplementary material for: Chromatin-accessibility estimation from single-cell ATAC-seq data with scOpen
Source: Nat Commun. 2021 Nov 4;12:6386. doi: 10.1038/s41467-021-26530-2 (PMC8568974; doi:10.1038/s41467-021-26530-2)
Supplement: Supplementary file 6 — Reporting Summary [file 41467_2021_26530_MOESM6_ESM.pdf]

nature research

Corresponding author(s): Ivan Gesteira Costa Filho

Last updated by author(s): Sep 22, 2021

Reporting Summary

Nature Research wishes to improve the reproducibility of the work that we publish. This form provides structure for consistency and transparency in reporting. For further information on Nature Research policies, see Authors & Referees and the Editorial Policy Checklist.

Statistics

For all statistical analyses, confirm that the following items are present in the figure legend, table legend, main text, or Methods section.

n/a	Confirmed	
☐	☒	The exact sample size (n) for each experimental group/condition, given as a discrete number and unit of measurement
☐	☒	A statement on whether measurements were taken from distinct samples or whether the same sample was measured repeatedly
☐	☒	The statistical test(s) used AND whether they are one- or two-sided<br>*Only common tests should be described solely by name; describe more complex techniques in the Methods section.*
☐	☒	A description of all covariates tested
☐	☒	A description of any assumptions or corrections, such as tests of normality and adjustment for multiple comparisons
☐	☒	A full description of the statistical parameters including central tendency (e.g. means) or other basic estimates (e.g. regression coefficient) AND variation (e.g. standard deviation) or associated estimates of uncertainty (e.g. confidence intervals)
☐	☒	For null hypothesis testing, the test statistic (e.g. F, t, r) with confidence intervals, effect sizes, degrees of freedom and P value noted<br>*Give P values as exact values whenever suitable.*
☒	☐	For Bayesian analysis, information on the choice of priors and Markov chain Monte Carlo settings
☒	☐	For hierarchical and complex designs, identification of the appropriate level for tests and full reporting of outcomes
☐	☒	Estimates of effect sizes (e.g. Cohen's d, Pearson's r), indicating how they were calculated

Our web collection on statistics for biologists contains articles on many of the points above.

Software and code

Policy information about availability of computer code

Data collection	Fluorescent and brightfield images were acquired using Nikon software (NIS-Elements AR 5.11.03) on Nikon A1R confocal microscope using 40X and 60X objectives (Nikon). RT-qPCR data was acquired on Bio-Rad CFX96 Real Time System (Bio-Rad). Other Software used: R, Python3, Graphpad (Version 8.4.3), ImageJ (1.53),. Sequencing was performed on the NextSeq Illumina sequencers at RWTH Aachen University Hospital.
Data analysis	Custom code used in this study is made available at https://github.com/CostaLab/scopen (https://zenodo.org/record/5513693),). All scripts for reproducing the analysis are available at https://github.com/CostaLab/scopen-reproducibility, as well as tables with all benchmarking results and raw count matrices from benchmarking datasets. Tutorial for the use of HINT-ATAC with the hematopoietic data set is provided in https://www.regulatory-genomics.org/hint/tutorial-differential-footprints-on-scatac-seq/.<br><br>The software and versions used in this study are:Samtools (v1.1.1),bedtools (v2.30.0),RGT (v0.13.2),deeptools (v3.5.1), bowtie2 (v2.2.5) R (v4.0.3), Python (v3.8.8), Seurat (v3), ArchR (1.0.1), MAGIC (v3.0.0) SAVER (v1.1.2), scImpute (v0.0.8), DCA (v0.3.1), cisTopic (v2.1.0) scBFA (v1.0), SCALE (v1.1.0), missMDA (v1.18), SnapATAC (v2.0), Cicero (v1.3.0), chromVAR (v1.14.0), scABC (v0.1)

For manuscripts utilizing custom algorithms or software that are central to the research but not yet described in published literature, software must be made available to editors/reviewers. We strongly encourage code deposition in a community repository (e.g. GitHub). See the Nature Research guidelines for submitting code & software for further information.

Data

Policy information about availability of data

All manuscripts must include a data availability statement. This statement should provide the following information, where applicable:

- Accession codes, unique identifiers, or web links for publicly available datasets
- A list of figures that have associated raw data
- A description of any restrictions on data availability

The scATAC-seq data generated from UUO mouse kidney and the RNA-seq data generated from cell culture experiments have been deposited in NCBI's Gene

Expression Omnibus and are accessible through GEO Series accession number GSE139950.

Publically available data used in this study includes: scATAC-seq for human primary blood cell (GSE74912), scATAC-seq for cell lines (GSE65360), scATAC-seq for hematopoiesis (GSE96769), scATAC-seq for T-cell (GSE107816), scATAC-seq for PBMC (ftp://ftp.ebi.ac.uk/pub/databases/mofa/10x_rna_atac_vignette/seurat.rds) scATAC-seq matrix for GM12878 (GSM2970932), Hi-C data for GM12878 (GSE81503), ChIA-PET data for GM12878 (GSM1872887) and H3K27ac and H3K4me1 ChIP-seq for human heart from ENCODE (ENCSR000CDG and ENCSR436FYE).

Field-specific reporting

Please select the one below that is the best fit for your research. If you are not sure, read the appropriate sections before making your selection.

☒ Life sciences ☐ Behavioural & social sciences ☐ Ecological, evolutionary & environmental sciences

For a reference copy of the document with all sections, see nature.com/documents/nr-reporting-summary-flat.pdf

Life sciences study design

All studies must disclose on these points even when the disclosure is negative.

Sample size	We measured snATAC-Seq data from 6 mice: 2 biological replicates for controls, day 3 and day 7 after UOO. RNA-seq experiments were based on 3 biological replicates. RT-qPCR and histological analysis were performed on multiple independent biological replicates with a minimum of 3 (n=shown in the figure and described in legends). No statistical methods were used to predetermine sample size.
Data exclusions	No data was excluded.
Replication	The reported findings were replicated across multiple biological samples (n reported in each Figure or Methods). Immunofluorescenct imaging was performed on 3 kidney samples minimum and repeated with similar results successfully. Positive and negative controls were done once per used sample.
Randomization	Randomisation was not relevant because every sample consisted of cells of the same cell types taken into experiment from the same biological and growth conditions.
Blinding	Investigators were blinded during imaging. Tissue collections for gene expression and protein analyses were not performed blind.

Reporting for specific materials, systems and methods

We require information from authors about some types of materials, experimental systems and methods used in many studies. Here, indicate whether each material, system or method listed is relevant to your study. If you are not sure if a list item applies to your research, read the appropriate section before selecting a response.

Materials & experimental systems

n/a	Involved in the study	
☐	☒	Antibodies
☐	☒	Eukaryotic cell lines
☒	☐	Palaeontology
☐	☒	Animals and other organisms
☐	☒	Human research participants
☒	☐	Clinical data

Methods

n/a	Involved in the study	
☒	☐	ChIP-seq
☒	☐	Flow cytometry
☒	☐	MRI-based neuroimaging

Antibodies

Antibodies used	The following antibodies were used: (vendor, name, catalog number, Lot, antibody dilution)<br>- Sigma-Aldrich, anti-RUNX1, HPA004176, Lot I104232 polyclonal, 1:100<br>- R&D, anti-PDGFRb, MAB1263, PR7212, Lot BQF0715061, 1:100<br>- Jackson ImmunoResearch, donkey anti-rabbit, 711-605-152, Lot 138314, 1:200
Validation	All antibodies used in this study are commercially avaiable. They are validated by the vendors for the specific assay and species used. The validation is available on the vendors website. Primary antibodies for immunostaining were validated by performing comparisons to species-matched isotype antibodies and unstained controls. The validation is available on the vendors website:<br><br>-https://www.sigmaaldrich.com/DE/de/product/sigma/hpa004176<br><br>-anti-PDGFRb, R&D, https://www.rndsystems.com/products/human-pdgf-rbeta-antibody-pr7212_mab1263

Eukaryotic cell lines

Policy information about cell lines

Cell line source(s)	The immortalized human PDGFRb+ kidney cell line from healthy tumor nephrectomy tissue was generated as described in the methods. The local ethics committee of the University Hospital RWTH Aachen approved all human tissue protocols (EK-016/17).
Authentication	None of the cell lines used were authenticated.
Mycoplasma contamination	Mycoplasma tests showed no evidende of contamination by PCR.
Commonly misidentified lines (See ICLAC register)	No commonly misidentified cell lines were used.

Animals and other organisms

Policy information about studies involving animals; ARRIVE guidelines recommended for reporting animal research

Laboratory animals	This study involved trangenic mice (Mus musculus). C57BL/6 male mice (age 8-10 weeks) and dgfrb-BAC-eGFP reporter female and male mice (age 8-12 weeks) by N. Heintz (The Rockefeller University) were housed two to five animals per cage with a 12-h light–dark cycle (lights on from 0700 to 1900 h) at sustained temperature (20 °C±0.5°C) and humidity (~50%±10%) with ad libitum access to food and water.
Wild animals	This study did not involve any wild animals.
Field-collected samples	This study did not involve any samples collected from the field.
Ethics oversight	Animal experiment protocols were approved by the LANUV-NRW, Germany. All animal experiments were carried out in accordance with their guidelines.

Note that full information on the approval of the study protocol must also be provided in the manuscript.

Human research participants

Policy information about studies involving human research participants

Population characteristics	Human kidney tissue samples for the generation of the primary human PDGFRb+ were generated from a male, 50-60 year old patient undergoing nephrectomy due to kidney cancer. Ethnicity was caucasian.
Recruitment	Healthy non-tumor kidney tissue was obtained intraoperatively from the patients undergoing surgical renal cell carcinoma resection at the Urology Unit of the University Hospital Eschweiler. All tissue samples used for this study were obtained with written informed consent from all patients in accordance with the guidelines of The Declaration of Helsinki 2000.
Ethics oversight	The local ethics committee of the University Hospital RWTH Aachen approved all human tissue protocols (EK-016/17).

Note that full information on the approval of the study protocol must also be provided in the manuscript.
